# Host Immune Regulation in Implant-Associated Infection (IAI): What Does the Current Evidence Provide Us to Prevent or Treat IAI?

**DOI:** 10.3390/bioengineering10030356

**Published:** 2023-03-13

**Authors:** Zulipikaer Maimaiti, Zhuo Li, Chi Xu, Jun Fu, Li-Bo Hao, Ji-Ying Chen, Wei Chai

**Affiliations:** 1 Department of Orthopaedics, The Fourth Medical Centre, Chinese PLA General Hospital, Beijing 100048, China; 2 Department of Orthopaedics, The First Medical Centre, Chinese PLA General Hospital, Beijing 100853, China; 3 School of Medicine, Nankai University, Tianjin 300071, China

**Keywords:** implant-associated infection, biofilm, host, immune modulation, nanomaterial, antibacterial

## Abstract

The number of orthopedic implants for bone fixation and joint arthroplasty has been steadily increasing over the past few years. However, implant-associated infection (IAI), a major complication in orthopedic surgery, impacts the quality of life and causes a substantial economic burden on patients and societies. While research and study on IAI have received increasing attention in recent years, the failure rate of IAI has still not decreased significantly. This is related to microbial biofilms and their inherent antibiotic resistance, as well as the various mechanisms by which bacteria evade host immunity, resulting in difficulties in diagnosing and treating IAIs. Hence, a better understanding of the complex interactions between biofilms, implants, and host immunity is necessary to develop new strategies for preventing and controlling these infections. This review first discusses the challenges in diagnosing and treating IAI, followed by an extensive review of the direct effects of orthopedic implants, host immune function, pathogenic bacteria, and biofilms. Finally, several promising preventive or therapeutic alternatives are presented, with the hope of mitigating or eliminating the threat of antibiotic resistance and refractory biofilms in IAI.

## 1. Introduction

An increasing number of orthopedic implants, including artificial joint prostheses and fracture fixation devices, such as nails, screws, and plates, are used in modern orthopedic surgical procedures [1]. Implant-associated infection (IAI) includes fracture-related infection (FRI) and periprosthetic joint infection (PJI) as the most undesirable complications after implantation. Not only do these infections present severe clinical problems, they also place serious economic burdens on patients as well as healthcare systems [2,3]. The incidence of PJI is estimated to be between 3% and 5%, with a recurrence rate of up to 15% after infection treatment. For patients with FRI, the recurrence rate is around 6–9%, with approximately 5% of patients requiring limb amputation [2,4,5]. According to earlier reports, the annual cost of treating all IAIs in the United States was approximately USD 3.3 billion (USD 1.86 billion for orthopedic IAI alone), and IAI accounts for 25.6% of all healthcare-associated infections [4,6]. Current data suggest that the annual hospital costs of PJI are estimated to reach USD 1.85 billion by 2030 in the United States [7]. Despite upgrades in implant materials and advances in surgical techniques, the number of patients with IAI is expected to increase as the population ages and complications leading to immunocompromised states become more common [8].

Various low-virulent pathogens can cause IAI, and the number of bacteria needed to cause IAI is significantly lower than in the case of no implant. The effectiveness of local immune defenses may also be a contributing factor. Additionally, pathogenic bacteria form biofilms on the surfaces of prostheses, which facilitate the escape of bacteria from host immune cells and protect them from antibiotic clearance [6,9]. Biofilm pathogens are a hundred times or even a thousand times more resistant to antibacterial agents than planktonic bacteria, which increases the challenge of treating IAI [10,11]. Therefore, the clinical treatment of IAI is more aggressive, with the main treatment modalities being the removal of the implant, extensive debridement, and antibiotic suppression therapy [12]. There is, therefore, an urgent need to find an alternative approach to improve conventional antibiotic treatment.

The immune response involving the host, the implant, and pathogenic bacteria during IAI is more complex than in cases without implants [6]. However, the immune microenvironment in IAI is not fully understood. Intervening in the host immune response and enhancing immune system defenses may prevent bacteria from evading innate and adaptive host defenses. Host immune modulation may offer new hope for the effective treatment of IAI. This review discusses the latest advances in this field and offers new prospects for preventing and treating IAI.

## 2. Challenges in the Diagnosis and Treatment of IAI

IAI is a catastrophic complication that orthopedic surgeons continue to face, and its prevention, diagnosis, and treatment remain challenging. Despite significant improvements in perioperative patient management, surgical techniques, and upgrades in surgical equipment and prosthetic materials in recent years, the incidence of PJI has not decreased significantly or even changed [13]. While many new diagnostic markers have been discovered and new clinical practice guidelines have been proposed, such as the 2018 International Consensus Meeting (ICM) criteria and the 2021 European Bone and Joint Infection Society (EBJIS) criteria for the diagnosis of PJI, accurate diagnosis still poses difficulties. Currently, no single test is 100% accurate, and all of these criteria consist of various clinical examinations, including laboratory tests, microbiological cultures, histopathology, and intraoperative findings [13,14,15,16].

The management of both PJI and FRI is based on debridement, antimicrobial therapy, implant retention (DAIR), or implant removal combined with antimicrobial therapy. Once infection is confirmed, surgeons must choose the best surgical option (DAIR, one- or two-stage revision). The ultimate goal of the surgical strategy is to remove all foreign material and dead tissues to reduce biofilm load. Unfortunately, there is always a proportion of patients who experience re-infection despite standard surgical and antibiotic treatment. Success rates of DAIR are around 84% [17,18,19,20], while success rates for one- or two-stage revision are generally comparable, ranging from 77% to 84% [21,22,23]. This also indicates that PJI patients who undergo complete infection-control procedures have a re-failure rate of approximately 15–20%. These patients experience multiple surgeries and prolonged antibiotic therapy, and their quality of life is severely affected. Some patients face worse outcomes, such as amputation or even death. Moreover, the overuse or inappropriate use of antibiotics in PJI treatment has led to increased bacterial resistance and, more problematically, infections caused by multidrug-resistant bacteria that can cause further complications [24].

## 3. The Role of Host Immunity in IAI

The relationship between implants, biofilms, and host immunity is complex, and a thorough understanding of these interactions is necessary for the development of effective countermeasures. In the following section, we briefly discuss the impact of implants on the host, the formation and development of biofilms and their significance in IAI, and immune evasion in biofilm infections.

### 3.1. Impact of Prostheses on Host Immune Status

Most orthopedic implants are permanently implanted into the patient’s body, and the host can sense the prosthetic material as foreign and create a specific immune-response microenvironment [25,26]. This sequence, called the fiber cascade, includes tissue injury, immune recruitment and adhesion, myofibroblast induction, and fiber capsule formation [27]. The presence of a fibrotic cascade reaction in this process may prevent interactions with the surrounding environment, including the sensing of biochemical stimuli, such as pH, oxygen, and the exchange of nutrients [28]. Tissue damage caused by biomaterial implantation leads to the immediate activation of the coagulation cascade response and subsequent initiation of innate immunity [29]. Due to tissue damage caused by surgery or injection, the local endogenous injury-related molecular model (DAMP) is activated. This process also involves the coagulation cascade, complement system, platelets, and immune cells—neutrophils which are the first immune cells to respond to an implanted biomaterial [30,31].

Additionally, damaged epithelial/endothelial cells and activated platelets secrete many cytokines. Several studies have reported differences in cytokine levels, including IL-6, TNF-a, IL-10, G-CSF, and CCL2, after joint prosthesis implantation, indicating that prostheses affect host immunity [32,33,34]. A recent study found that several soluble immunoregulatory markers (sCTLA-4, sPD-1, sPD-L1, sPD-L2, sTIM-3, and sLAG-3) from hip and knee aspirates in total joint arthroplasty (TJA) patients have higher mean concentrations than native joints [35]. Animal models of orthopedic-implant-associated infections have also demonstrated that the phagocytic and bactericidal activities of polymorphonuclear leukocytes (PMNLs) are decreased after foreign-body implantation [36]. This may be due to a complex PMN defect induced by the interaction of PMNs with non-phagocytic foreign bodies, which leads to high susceptibility to infection by foreign bodies [37]. Implants also reduce bacterial recognition and phagocytosis, decreasing the host’s clearance of planktonic bacteria and thus reducing the number of bacteria required for infection [38]. Ultra-high-molecular-weight polyethylene (UHMWP) particles can impair local neutrophil function, leading to reduced bacterial killing by neutrophils. Bernard et al. demonstrated that the presence of UHMWP particles impairs neutrophil bactericidal activity, which explains the susceptibility of loose implants to bacterial infections [39].

### 3.2. Biofilm Formation on Implants

The definition of bacterial biofilms is three-dimensional multicellular communities of one or several species protected from the outside environment through the production of a matrix. The biofilm matrix is composed of extracellular polymeric substances (EPSs), and EPSs consist of proteins, exopolysaccharides, extracellular DNA (eDNA), teichoic acids, and lipids [9,40,41]. This EPS matrix enables the species to attach to a biotic or an abiotic surface for survival and further establishment. Gram-positive organisms, such as Staphylococcus aureus (*S. aureus*), enterococci, and coagulase-negative Staphylococci (CoNS), and Gram-negative organisms, including Pseudomonas and Serratia, are the most common microorganisms in implant-related chronic infections [42]. PJI is primarily caused by *S. aureus* and CoNS infections, which account for about 50% of PJIs [11,43,44,45]. In comparison, streptococci and enterococci account for about 10% of cases. These bacteria can be protected by the biofilm that forms on the implant’s surface; biofilm formation is essential for survival against antibiotics and in eliminating immune cells.

**Figure 1:**
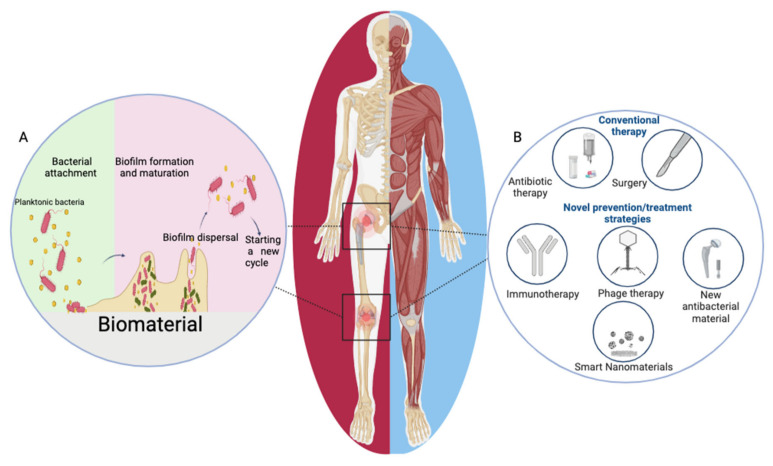
(**A**) The process of biofilm formation: (1) bacteria approaching and irreversibly attaching onto the implant surface, (2) biofilm formation and maturation, (3) biofilm dispersal, and (4) start of a new cycle. (**B**) Conventional therapy and novel prevention or treatment strategies: (1) immunotherapy, (2) phage therapy, and (3) use of new antibacterial materials and (4) smart nanomaterials.

Biofilm establishment exists in different phenotypes depending on the type of bacteria and the surrounding environment, usually ranging from reversible attachment to the surface of the prosthesis to irreversible colonization forming a multicellular community (Figure 1A). Biofilm formation involves several steps: first, adhesion to the surface of the prosthesis, followed by cell aggregation and production of EPS, organization into microcolonies, further remodeling, and eventual maturation into large colonies. Bacteria will disperse from the mature biofilm and then spread to other places, establishing new biofilms [6,41,46,47]. The biofilm formed on the implant’s surface protects the bacteria. It promotes the persistence of the infection so that the implant-infected bacteria can evade inherent and adaptive host defenses and biocidal and antibiotic agents applied in treatment.

Biofilms are significantly less sensitive to antimicrobial agents than non-adherent planktonic cells, and the biofilm phenotype was found in more than 80% of non-acute infections. Its critical features were reported as considerable resistance to environmental stresses, antimicrobials, disinfectants, and host immune defenses [48]. The various effects of antimicrobial agent interactions with biofilm matrix components reduced growth rates, and specific genetic determinants of antibiotic resistance and tolerance contribute to the characteristically high degree of recalcitrance observed in biofilm communities [49]. Antibiotic resistance is closely related to biofilm properties, including the presence of an oxygen and nutrient gradient across the biofilm, resulting in reduced metabolic activity and prolonged bacterial proliferation, causing bacteria to become dormant. In addition, biofilm growth is associated with increased levels of mutations and quorum-sensing regulatory mechanisms, chromosomal beta-lactamase, upregulated efflux pumps, and mutations in antibiotic target molecules in bacteria, which can increase antibiotic resistance [50].

### 3.3. Immune Evasion in Biofilm Infection

There are different explanations for the immune evasion mechanisms of the biofilms produced on the surfaces of prostheses. These mechanisms include recruiting myeloid-derived suppressor cells (MDSCs) and macrophage polarization toward an anti-inflammatory state [51]. Staphylococcal biofilms promote the anti-inflammatory properties of monocytes and macrophages through MDSCs and reprogram the host’s innate immune response [52,53,54]. Kristian SA et al. showed that S. epidermidis biofilm formation could interfere with the deposition of immunoglobulin G (IgG) and C3b on the bacterial surface, leading to diminished complement system activation and killing by PMNs [55]. In addition, *S. aureus* biofilms circumvent Toll-like receptor 2 (TLR2) and TLR9-mediated recognition and inhibition of macrophage phagocytosis, while *Pseudomonas aeruginosa* downregulates pathogen-associated molecular pattern expression during biofilm development and evades immune recognition [49,56]. A recent study by Heim et al. demonstrated that *S. aureus* biofilms produced D-lactate to inhibit HDAC11, reprogramming the host immune response during infection [54]. Quorum sensing (QS), by which bacteria sense and respond to cell density status, plays an essential role in the maturation of biofilms and is necessary for bacterial survival [57]. While He et al. found that in *S. aureus* QS dysfunctional mutations were only present in biofilm infections, QS-dysfunctional bacteria have a significant survival advantage in biofilm infections and provide resistance to phagocytic attack [58].

In addition to biofilm disruption of host immune function, as described above, inhibition of complement activation and the production of virulence factors to disrupt immune recognition also occur, leading to more chronic and persistent bacterial infections [51]. Post V et al. reported that the phenotypic and genetic characteristics of *S. aureus* strains differed significantly between PJI and non-implant-associated infections and presented specific genetic virulence patterns in certain staphylococcal protein A (SpA) types found only in isolates cultured from orthopedic-implant-associated infections, with microbiological virulence factors differing between infection types [59]. Moreover, bacteria can hide in bone tissue, including osteoblasts, before producing biofilm, evading clearance by host immune cells [60].

## 4. Role of Main Host Immune Cells in IAI

### 4.1. Neutrophils and Macrophages

Neutrophils are classically considered as the first line of defense against invading pathogens [61]. Neutrophils recruited to the site of infection can kill bacteria through phagocytosis, degranulation of antimicrobial substances into the environment, and neutrophil extracellular trap (NET) formation [62]. Phagocytosis is a very effective strategy for neutrophils to eliminate planktonic bacteria or even small aggregates of bacteria, but biofilms are more resistant to neutrophil killing than planktonic bacteria, and phagocytosis efficiency seems to decrease with biofilm maturation [63,64]. Neutrophils secrete granulins, elastase, myeloperoxidase, and cytokines/chemokines (IL-1β, IL-6, IL-12, MCP-1, CXCL1, and CXCL2), and the production of reactive oxygen species (ROS) after a similar interaction with raw implant material was also observed in a mouse study model [65,66]. These cytokines generate various signals that attract monocytes and propagate the inflammatory response [65]. One of the mechanisms by which neutrophils create an inflammatory environment is the release of NETs, which consist of deoxyribonucleic acid and associated histones in neutrophil granules [67].

The formation of NETs is considered an effective strategy for capturing and clearing pathogenic microorganisms. The dysfunction of NETs leads to the release of cytotoxic factors causing damage to host tissues. Moreover, pathogens can respond to NETs in ways that circumvent the antimicrobial effects of NETs [68]. Abaricia et al. demonstrated that the co-culture of neutrophils and macrophages on a titanium surface in a mouse model induced pro-inflammatory macrophage polarization. Still, inhibition of NETosis enhanced anti-inflammatory macrophage polarization, suggesting that NETosis may be a therapeutic target [69]. Evidence suggests a direct or indirect interrelationship between neutrophils and other cells. Neutrophils secrete chemokines that aid in the recruitment of monocytes [70]. As demonstrated in a sterile inflammation model, macrophages can promote neutrophil recruitment and influence neutrophil activity [71]. Moreover, neutrophil production of ROS and disruption of the bactericidal mechanism of the NOS pathway decreased the killing capacity of neutrophils [72]. Yavari et al. viewed neutrophils as promising cellular targets in implant infection [29].

Macrophages are the critical line of defense against pathogens, and their activation mechanism determines how the host responds to pathogen attacks [73]. Macrophages accumulate gradually at the site of infected tissues against bacterial pathogens not cleared by PMNs [74]. Due to the differences in macrophage phenotypic polarization, bacterial elimination also has different effects. Polarized macrophages can be classified as classically activated (M1) or activated (M2) MΦs. M1 macrophages release pro-inflammatory cytokines, including IL-1β, IL-6, IL-12, and TNF-a, to mediate phagocytosis to eliminate bacteria. Conversely, activated M2 macrophages release anti-inflammatory cytokines il-4, IL-10, and arginine to reduce bactericidal responses [73,75,76,77]. Macrophages respond to planktonic bacteria and biofilms differently due to their response mechanisms. M1 macrophages promoting pro-inflammatory responses could effectively phagocytose single or planktonic stages of *S. aureus*. However, established biofilms have a dense polymeric matrix that is difficult to phagocytose by macrophages, and biofilm alters the host immune response [78]. Macrophages were found to polarize toward M2 macrophages to promote anti-inflammatory responses in biofilm-forming *S. aureus* infections, which may be related to the persistence of biofilm survival [51,53].

While the actual situation of macrophages during the progression of infection in vivo may be more complex than in vitro, macrophages’ role in implant infection needs further validation [79]. Studies have indicated that immunotherapy promotes the polarization of M1 macrophages by producing crucial drivers, such as interferon (IFN)-γ, TNF-α, and granulocyte-macrophage colony-stimulating factor (GM-CSF) [80,81,82]. The combination of direct bacterial cell killing and indirect immune cell recruitment enhances the adequate clearance of bacterial infections. Tacke R et al. developed a macrophage-based cell therapy that effectively killed Gram-positive and Gram-negative multidrug-resistant pathogens, and macrophage-based cell therapy was used as an adjunctive strategy for treating refractory bacterial infections [83].

### 4.2. Myeloid-Derived Suppressor Cells (MDSCs)

The importance of MDSCs in IAI has attracted widespread attention with the discovery of their potential value [84,85]. MDSCs originate from myeloid progenitor cells, immature myeloid cells (IMCs) that are generated in the bone marrow and which differentiate into mature granulocytes, macrophages, or dendritic cells (DCs) in healthy individuals [86]. In pathological conditions, such as cancer, various infectious diseases, trauma, bone marrow transplantation, and certain autoimmune diseases, IMCs are prevented from reaching the immature stage of differentiation. Those cell populations with a robust immunosuppressive effect on T cell responses are precisely MDSCs [87]. Based on cell-surface markers expression profiles, MDSCs are mainly divided into two subtypes, namely monocytic-MDSCs (M-MDSCs) and granulocytic MDSCs (G-MDSCs). MDSCs are vital players in balancing the inflammatory response and the pathogenesis of infection during infection [88]. However, some pathogens can exploit the suppressive immune effects of MDSCs to escape host immune clearance and promote the persistence and chronicity of infection [89].

Biofilm of *S. aureus* alters the mechanisms of host immune regulation, leading to the preferential accumulation of MDSCs and attenuation of the pro-inflammatory activity of macrophages [90]. The role of MDSCs in IAIs has been described in a series of studies by Heim and his colleagues [52]. In biofilm infections caused by *S. aureus*, MDSCs increased the expression of the inhibitory T cell-proliferation-associated factors arginase-1 and IL-10 [52]. A significant increase in MDSCs at the site of infection was observed in both PJI patients and mouse PJI models, while monocytes, macrophages, and T cells were reduced to varying degrees. MDSC-proliferation-associated cytokines IL-12, IL-1β, TNF-α, and G-CSF and the chemokines CXCL2 and CCL5 were also significantly elevated [91]. Recent studies have shown significant differences in immune cell distribution between PJI and non-infected samples, with an increase in M-MDSC/non-granulocyte ratios in PJI patients, further confirming the relevance of MDSCs in PJI [92]. MDSCs are a critical factor in the chronicity of *S. aureus* biofilm infections because their immunosuppressive function distorts the host’s immune response toward anti-inflammation. MDSC-targeted drugs may inhibit the function of MDSCs, and targeted modulation of MDSCs may be a new tool for preventing and treating IAI.

### 4.3. T Lymphocytes and Other Immune Cells

T lymphocytes are essential components of the adaptive immune system, most notably in T-cell reduction during acute and chronic inflammation (including trauma, sepsis, infection, and cancer), including helper T-cell and cytotoxic T-cell subsets. However, the role of T cells in the immune response against IAI has not been fully determined. In animal infection models, initial upregulation of antibodies to the *S. aureus* biofilm-specific antigen Th1 and concomitant upregulation of Th1- and Th17-related cytokines, including elevated systemic IL-6 levels, were observed [47,93,94]. Th2 antibodies were not upregulated until late in the infection, and the Th2/Treg response was protective against chronic *S. aureus* infection [95]. In a rat implant infection model, reduced systemic immune responses were observed in IAI rats compared to those without infection and were associated with diminished macrophage infiltration, increased levels of MDSCs in local soft tissues with a significant systemic reduction in T cells, and also MDSCs using various mechanistic pathways to stimulate immunosuppressive Treg activity [96]. Immunotherapies targeting various immune cells, including MDSC and T cells, have shown promising therapeutic potential in areas such as cancer and are expected to be used in other diseases associated with immunosuppression, including IAI [96,97].

In addition to T cells, B cells and NK cells play essential roles in infection. Like macrophages, B cells are among the specialized antigen-presenting cells (APCs) that efficiently recognize natural protein antigens on the surfaces of pathogens, generating antigen fragments that can be loaded into major histocompatibility complex (MHC) molecules, which then present the antigen fragments to T cells for recognition [98]. Vantucci et al. found that in IAI rats the number of B cells increased in the infected group and peaked at day 7, in contrast to the control group, in which B cell levels remained relatively stable [96]. The mechanisms of B-cell response during *S. aureus* infection and how the humoral immune response is skillfully evaded are summarized in a review by Muthukrishnan et al. *S. aureus* regulates B-cell survival and function by producing SpA [99]. During infection, it binds in the wrong direction to the Fcγ structural domain of immunoglobulins(Ig) and crosslinks the Fab domain of V_H_3-type B cell receptor IgM, allowing *S. aureus* to evade antibody detection and antibody-mediated phagocytosis [100,101].

NK cells release cytotoxic molecules, including perforin, granzyme, and granulosa, all of which have strong antibacterial effects against various types of bacteria [102,103,104]. In addition, crosstalk between NK cells and other immune cells, such as DCs and macrophages, allows them to play an antimicrobial role in the infection process [105]. Korn et al. compared the joint fluid of infected and non-infected patients by flow cytometric assay, and they found that the proportion of NK cells in the joint fluid of infected patients was significantly lower than in that of the non-infected group [92].

Moreover, alterations in the number and function of DCs have been reported in infections caused by microorganisms such as *S. aureus*. In human patients, T cells were found at the site of infection with biological material, and the activation and regulation of these CD4+ Th cells were regulated by DCs [106]. Methicillin-resistant *S. aureus* (MRSA) evades the host’s innate and acquired immune system by expressing multiple virulence factors (Panton–Valentine Leukocidin (PVL), α-toxin, and phenol-soluble modulin (PSM) peptides) [107]. The virulence factor PSMs produced by *S. aureus* interfere with adaptive immunity by modulating DC subpopulations in vivo [108]. Balraadjsing et al. demonstrated DC-mediated T cell proliferation and Th1/Th2 cell development in an in vitro model of biomaterial-associated infected cells [109]. They confirmed that *S. aureus* induced DC cytokine secretion, T cell proliferation, and Th1 cell formation better than *S. epidermidis*. In conclusion, bacterial species affect DC-dominated immune regulation in implant-associated infections differently. Various types of immune cells play different essential roles in IAI, and their interrelationships are complex; their roles in the local and systemic immune environment need to be explored in more detail.

## 5. Prevention and Treatment of IAI

### 5.1. Antibacterial Materials and Immune-Evasive Coatings for Orthopedic Implants

Antibacterial material use or the surface modification of implants can effectively inhibit biofilm formation on implant materials, providing a way to reduce the susceptibility of implants to infection (Figure 1B). Titanium (Ti) alloys, tantalum (Ta) alloys and cobalt (Co) alloys have been widely used in orthopedics, and all of them have shown good anti-microbial properties. Ti-Nb-Ta-Zr alloy, Ti-Mo alloy, Ti-Al-Sr-Zr alloy, and CoCrWNi alloy are under development, and new alloy materials are expected to be effective in preventing IAI by adding antibacterial elements, such as Cu, Ag, and Ga, based on previous research [110]. Ta alloys, which are also frequently used in orthopedics, have good biocompatibility, excellent mechanical properties, and strong corrosion resistance. Studies have reported that Ta nanofilms enhance the phagocytosis of bacteria by neutrophils, reduce the lysis of neutrophils, and promote the release of pro-inflammatory cytokines by macrophages, and this immunomodulatory effect helps the host to eliminate bacteria [111]. It has been confirmed that Ta can exhibit excellent antimicrobial activity because Ta surfaces can inhibit ATP synthesis, promote reactive oxygen species (ROS) generation, lead to cell membrane lipid peroxidation, reduce catalase activity and glutathione levels, and downregulate the expression of bacterial virulence factors, which are associated with the adhesion and viability of bacteria [112]. New antimicrobial metals and alloys can further improve the antimicrobial properties of implanted materials. However, the effects of new antimicrobial alloys on host immunity are still unknown, and the mechanisms need to be further clarified.

In the last few years, many studies on implant surface topographical engineering, including anti-fouling, quorum-sensing interfering surfaces, bactericidal implant surface topographical engineering, and the production of surface coatings through chemical modifications, have been conducted and advances have been made. Studies have demonstrated that bactericidal surfaces engineered with topographical features or stably tethered antibiotics or antimicrobial peptides can reduce bacterial adhesion, colonization and growth, and biofilm formation on implant surfaces [113]. More recently, Chae et al. developed an advanced surface modification technique for orthopedic implants termed the lubricated orthopedic implant surface (LOIS) method. A LOIS has a durable, extreme liquid repellency against a wide range of cells, proteins, calcium, and bacteria, along with antimicrobial properties and mechanical durability [114]. Although not yet widely used in the clinic, these techniques could one day be part of an effective strategy for preventing IAI and reducing the risk of infection.

### 5.2. Nanomaterials in IAI Prevention

Targeted delivery of anticancer drugs to target cells in cancer therapy research has inspired more precise and targeted delivery of existing antimicrobial agents through the use of engineered nanostructured materials which can bypass the barriers that prevent traditional pharmacological approaches [115,116]. Some recent studies involving biofilm elimination have shown that nanoparticles can kill bacteria directly or release biocides that disrupt biofilm formation, thereby reducing microbial survival [117,118]. Hong et al. discussed smart nanomaterials, including magnetic-responsive nanomaterials, light-responsive nanomaterials, pH-responsive nanomaterials, and enzyme/toxin-responsive nanomaterials, which are ideal for targeted therapeutic implant biofilms, which can control release of an antibacterial drug or exert antibacterial action under endogenous stimuli or external stimuli [119]. Therefore, nanomaterials are expected to be another means of overcoming implant biofilm infection (Figure 1B). Studies have confirmed that nanomaterials can influence macrophage polarization. Metal nanoparticles, such as Au nanoparticles and Ag nanoparticles, can induce M1 polarization, and smaller metal nanoparticles were more effective in inducing M1 macrophage polarization than large nanoparticles [120,121]. However, the interaction between nanomaterials and host immune response in IAI has not been extensively studied. Moreover, the clinical application of nanomaterial treatments for IAI is still some time away, as most of the work is still in the research stage.

### 5.3. Phage Therapy in IAI

With the growing concern of antibiotic resistance, phage therapy has started to attract public attention. Phages exhibit proven antimicrobial activity against multidrug-resistant pathogens and can act on biofilms, so phages are expected to be an alternative or adjunctive therapeutic option in implant infections (Figure 1B). Genevière et al. systematically reviewed nearly 20 years of clinical studies related to phage therapy in bone and joint infections, and phage use alone or in combination with antibiotics appears to be effective and safe in bone and joint infections [122]. Recent phage therapy in IAI studies demonstrated antibacterial effects in MRSA and Pseudomonas aeruginosa implant infection models [123]. An in vitro study using a phage cocktail consisting of a combination of five phages with bactericidal activity against *S. aureus* significantly reduced the number of bacteria in biofilms on porous Ti surfaces [124].

In addition, the synergistic mechanism of action between phages, antibiotics, and immune response includes phage-mediated degradation of pods or biofilms, which allows antibiotics, antibodies, complement systems, and phagocytosis to function [125].

In contrast to conventional antibiotics, phages target the lysis of host cells and have no or a minimal effect on the gut microbiota, which also avoids the occurrence of adverse events due to gut flora disorders [126]. Given the promising antimicrobial effects demonstrated by phages in animal and in vitro studies, phage therapy may be a new alternative to conventional antimicrobial therapies. Although phage therapy has shown potential therapeutic value, randomized clinical trials are necessary to evaluate the efficacy of phages in IAIs.

### 5.4. Immune Modulation in IAI

The challenges in IAI urgently require us to explore other viable alternative therapeutic approaches combined with conventional antibiotic treatments to reduce the disease burden threatened by antibiotic-resistant pathogens. Studies have shown that immune evasion to resist host immune clearance is a main feature of bacterial infection [6,63,99]. Recently, more and more studies have discussed the possibility of immunotherapy in IAI (Figure 1B). Improving understanding of the relationship between pathogens, implants, and the host immune system and discovering therapeutic targets for systemic immunomodulation may improve the ability to combat biomaterial-associated infections and improve patient prognosis [97,127]. In the following sections, we will briefly describe immunomodulatory options besides the targeted modulation of host immune cells (as mentioned above).

#### 5.4.1. Monoclonal Antibodies (mAbs)

New studies have demonstrated that mAbs could provide a potential therapeutic/diagnostic approach for biofilm-associated infections [127,128]. Biofilm-binding monoclonal antibodies can act as delivery vehicles to specifically carry radionuclides or biofilm-degrading enzymes, antibiotics, and photosensitizers to the site of infection for diagnostic or therapeutic purposes [128]. For therapeutic purposes, studies have confirmed that antibodies can potentially interfere with biofilm formation or the spread of established biofilms through various mechanisms [129]. The implant material is encapsulated in the body by serum and various tissue proteins containing collagen and fibronectin. Bacteria recognize the above host proteins by microbial surface components recognizing adhesive matrix molecules (MSCRAMMs) and specifically bind and irreversibly adhere to the implant surface [6,97]. SpA is one of the MSCRAMMs and a potential target for immunotherapeutic approaches against *S. aureus* infections. It was shown that a protective monoclonal antibody (2H7) targeting the conserved domain of SpA against MRSA infection in models of sepsis and peritoneal infection showed significant protection [130]. Varshney et al. demonstrated that monoclonal antibody 514G3, which targets SpA, promotes natural immune facilitation of clearance of MRSA and MSSA strains [131]. Yokogawa et al. found that anti-glucosaminidase monoclonal antibodies (anti-Gmd) not only cleared staphylococcal colonies and inhibited dissemination but also reduced the local inflammatory response, thereby reducing bone loss; in addition, anti-Gmd in combination with vancomycin was more effective in preventing re-infection [132]. Monoclonal antibody therapy in IAI is a promising alternative, but most trials have been performed on animal models and evaluation of IAI infection in humans is still lacking.

#### 5.4.2. Immune Checkpoint Molecules

Immune checkpoint molecules are modulator receptors expressed on immune cells that trigger immunosuppressive signals [133]. In chronic infections or cancers, sustained antigen exposure results in the continued expression of PD-1, which may impede immune-mediated clearance of pathogens, degenerating cells, and tissue damage [134]. CTLA-4, also expressed in T cells, interacts with CD80/CD86, limiting T cell activation [135]. Due to the critical role of immune checkpoints in suppressing effector T-cell responses, they have become targets for cancer treatment. Studies have demonstrated that immune checkpoint inhibitors enhance ex vivo effector T cell responses in patients with chronic viral, bacterial, and parasitic infections [136]. Studies have shown that PD-1/PD-L1 is involved in sepsis-induced immunosuppression and that increased levels of PD-1 expression in patients with sepsis increase the risk of secondary infection [137]. Treatment with PD-1 and PD-L1 antibodies in animal models of sepsis with different pathogens, such as bacteria and fungi, effectively improved survival rates [138]. Beyond PD-1/PD-L1 and CTLA-4, ligands targeting the “new-generation” immune checkpoint T cell immunoglobulin- and mucin-domain-containing molecule 3 (TIM-3), lymphocyte-activation gene-3(LAG-3), T cell immunoreceptor with immunoglobulin and immunoreceptor tyrosine-based inhibitory motif domain (TIGIT), and inhibitory ligands in the B7 family (B7-H3, B7S1, and VISTA) are also significant targets in cancer therapy and potential research foci in the context of pathogenic infections [139].

Qualitative and quantitative analysis is required for immunomodulatory molecules to understand the immune system’s role in IAI. In a recent study, Jubel et al. evaluated the concentrations of soluble immunoregulatory molecules in PJI and aseptic loosening patients; they examined a total of 14 soluble immunoregulatory markers in patients’ joint fluid and concluded that nine soluble markers, including sLAG-3, sCTLA-4, sCD27, sCD80, sCD28, sTIM-3, sPD-1, IDO, and sBTLA, were significantly different in PJI patients compared to controls [35]. The potential value of immune checkpoint molecules in preventing or treating IAI still needs further exploration, as relevant studies are currently lacking.

#### 5.4.3. Cytokine Modulation

Cytokines are essential messenger molecules that mediate immune cell functions, and cytokine signaling modulation may effectively treat many bacterial infections [140]. Cytokines such as IL-1α, IL-1β, and TNF-α participate in pathological processes, such as chronic inflammation, autoimmunity, and malignant diseases. Thus, the critical role of cytokines in foreign device implantation and IAI is gaining attention. Cytokines play a crucial role in bacterial infections and exhibit different effects. Cytokine production may be beneficial for host survival in some cases, but in some situations it suppresses the immune response during bacterial infections, preventing proper clearance of the bacteria and facilitating their persistence in the host [141].

In an IAI animal study, IL-1α, IL-5, IL-10, IL-12p70, IL-13, GM-CSF, IFN-γ, and TNF-α were found to be significantly elevated in the tissues of mice with implants and IAI, whereas IL-1β, IL-4, and IL-6 were explicitly elevated in mice with IAI [142]. Furthermore, another study that included 17 patients (6 primary total knee arthroplasty and 11 total knee revision patients) showed that implantation of the prosthesis caused a rise in six cytokines, including IL-10, IL-12p70, IL-13, IL-17A, IL-4, and TNF-α, while infection-specific cytokines increased in PJI patients included IL-1α, IL-1β, IL-6, IL-8, MCP-1, MIP-1α, and MIP-1β [143]. Vaudaux et al. confirmed that elevated local TNF levels might improve host defense against staphylococcal foreign-body infection [144]. This conclusion is supported by the study of Wang et al. TNF and IL-1β contribute to host defense against *S. aureus* IAI, and monocyte recruitment is mediated by both IL-1β and TNF [145]. Therapeutic manipulation of cytokines might play a key role in controlling the infection and reducing the inflammatory response caused by the infection. However, in orthopedics, especially in IAI, cytokines are mainly used for diagnostic purposes, and the regulatory control between cytokines and host cells, as well as their potential preventive/therapeutic value, still need to be explored.

## 6. Conclusions Remarks

IAI is a severe complication in orthopedic surgery, and there are still a lot of unknown mechanisms of host immunity involved in the development of IAI. A better understanding of the interactions between bacteria, host immunity, and implant materials will provide more promising preventative or therapeutic solutions. Thus, developing effective treatment strategies for IAIs is of the utmost importance. In terms of surgical intervention, debridement and removal of the infected implant remains a crucial aspect of treatment for most IAIs, especially in cases where the pathogen has formed mature biofilms. However, the timing of surgery is critical and must be considered in light of the patient’s immune status, comorbidities, and the severity of the infection. In addition to surgical intervention, the use of antimicrobial agents remains a cornerstone of IAI treatment. The choice of antimicrobial therapy should be guided by the results of bacterial culture and susceptibility testing. It is essential to ensure adequate dosing and duration of therapy to prevent the development of resistance. Moreover, strategies to enhance the immune response may be a promising avenue for future research. Immunomodulatory agents, such as interferons, may have a role in mitigating infections and reducing the risk of implant failure. This review has described the current research landscape related to IAI and provided a brief overview of several potential therapeutic approaches that have direct or indirect effects on host immunity and that hold promise for clinical application.
